# Preoperative Hyponatremia Indicates Complicated Acute Appendicitis

**DOI:** 10.1155/2022/1836754

**Published:** 2022-03-31

**Authors:** Nikolaos G. Symeonidis, Efstathios T. Pavlidis, Kyriakos K. Psarras, Kalliopi Stavrati, Christina Nikolaidou, Alexandra Marneri, Georgios Geropoulos, Maria Meitanidou, Emili Andreou, Theodoros E. Pavlidis

**Affiliations:** School of Medicine, Second Surgical Propedeutic Department, Ippokrateio General Hospital, Aristotle University of Thessaloniki, Konstantinoupoleos 49, Thessaloniki 546 42, Greece

## Abstract

**Introduction:**

Acute appendicitis is the most common surgical emergency. Early detection of patients with complicated appendicitis leads to prompt surgical management and better outcome. This study investigated the relationship between the severity of acute appendicitis and the presence of preoperative hyponatremia.

**Materials and Methods:**

We retrospectively reviewed the medical files of adult patients operated on for acute appendicitis over a 6-year period. Hyponatremia was defined as serum sodium level of ≤135 mEq/L. Patients were classified into complicated appendicitis and noncomplicated appendicitis according to operative findings and/or histopathology reports.

**Results:**

A total of 129 patients were identified and included in this study. Complicated appendicitis was found more frequently in female patients and older patients. Hyponatremia was found significantly more frequently in patients with complicated appendicitis (*p* < 0.001) and also in patients with perforation than without perforation (*p*=0.047).

**Conclusions:**

The present study demonstrated that preoperative hyponatremia is associated with complicated appendicitis. Serum sodium levels, a routine, low-cost laboratory test, could act as an accessory marker aiding surgeons in earlier identification of gangrenous or perforated acute appendicitis.

## 1. Introduction

Acute appendicitis is the most common cause of acute abdominal pain and the most common reason for emergency abdominal surgery around the world [1]. It is estimated that in the Western world, emergency appendectomy will be performed in 7-8% of the general population at some point during their lifetime [2]. Recent advances in imaging and treatment failed to significantly improve both morbidity and mortality [3]. In the majority of the cases, patients suffer from simple, noncomplicated appendicitis (NCA). When patients present with complicated appendicitis (CA), the postoperative outcome is significantly worse with increased morbidity, delayed recovery, and increased hospital costs [4].

Classification of patients diagnosed with acute appendicitis in the CA group is defined by the presence of a perforated or gangrenous appendix [5]. Perforation can occur in 20–30% of the cases, but higher rates are reported in both children and the elderly [6, 7]. Especially in children under 5 years of age, perforation rates are even higher, ranging from 100% in children younger than 1 year to 47.3% in children 5 years old [8]. While early surgery in CA is associated with fewer postoperative complications, delayed surgery results in longer hospital stay and increased costs [9]. Patients with appendiceal perforation presenting with diffuse peritonitis clearly need to be operated promptly, but defining the urgency for appendectomy for the rest of the patients can prove difficulty. Early recognition of patients with CA may affect decision-making regarding the timing of the surgery as well as the suitability of nonoperative management. Various scoring systems have been introduced in order to assess patients with acute appendicitis such as Alvarado, Pediatric Appendicitis Score (PAS), and the Appendicitis Inflammatory Response Score (AIR). None of these scoring systems has been proven to be of an adequate predictive value for the identification of CA in the adult population, highlighting the need for markers that have improved specificity and are easy to use in the emergency clinical setting [10].

Aiming to improve detection of CA, numerous studies have introduced a variety of demographic (age, gender, and comorbidities), clinical (fever, tachycardia, and symptom duration), and laboratory (C-reactive protein, leukocytosis, neutrophil count, neutrophil/lymphocyte ratio, erythrocyte sedimentation ratio, and bilirubin) parameters to be associated with CA [11, 12]. Hyponatremia (serum sodium level <135 mEq/L) is a frequent electrolyte disorder and is associated with increased perioperative morbidity and mortality [13]. Although the exact pathophysiological relationship between hyponatremia and severe inflammation is yet to be elucidated, several studies have demonstrated that the development of hyponatremia in severe inflammation cases is a process which probably involves proinflammatory cytokines such as interleukin- (IL-) 1*β* and IL-6 as well as antidiuretic hormone (ADH) secretion [14, 15].

Considering that early detection of cases with CA would benefit from prompt surgical management, we investigated the relationship between the severity of acute appendicitis and the presence of preoperative hyponatremia.

## 2. Materials and Methods

The present study was approved by the institutional scientific review board (ISRB number: 138/15-4-2020). We retrospectively reviewed the medical files of all patients (nonpediatric, aged over 14 years) operated on for acute appendicitis over a 6-year period (January 2014–December 2019). Patients with incomplete data, appendiceal neoplasms, appendicitis during pregnancy, and American Society of Anesthesiology (ASA) score ≥3 were excluded from the study. Study data included patient demographics such as age and gender, laboratory values on admission, preoperative imaging findings, operative notes, and histology notes. Patients were classified into 2 groups: the complicated appendicitis (CA) group and the noncomplicated appendicitis (NCA) group. The inclusion criteria of patients in the CA group were mostly based on the intraoperative findings of perforated or gangrenous appendix and the presence of intraabdominal periappendiceal abscess or peritonitis, as recorded in the patients' operative notes. Histological confirmation of transmural necrosis in cases in which the operative notes were unclear was also sought. Evidence of appendiceal perforation was used to further stratify the patients with CA into two subgroups (perforation–no perforation). Based on our institution's laboratory normal serum sodium levels, hyponatremia is defined as a serum sodium level ≤135 mEq/L and normonatremia as serum sodium level >135 mEq/L and ≤145 mEq/L.

The primary outcome of the study was the association between low serum sodium levels detected upon admission and the finding of complicated appendicitis and second, the presence of a perforated appendix. Secondary outcome was also the identification of parameters both preoperative (demographic characteristics and presenting symptoms) and postoperative (complications) also associated with complicated appendicitis.

Statistical analysis was performed with the use of IBM SPSS 13.0 (SPSS Inc., Chicago, IL, USA), and the statistical tests used were Student's *t*, Pearson's chi-square, and Fisher's exact, depending on the type of variables tested and sample size. The normality of the distribution was checked my means of the Shapiro–Wilk test. Assessment of the cutoff value for the prediction of CA was performed with the receiver operating characteristic curve (ROC) analysis, and the values of the area under the curve (AUC), sensitivity, and specificity were calculated as mean. The level of statistical significance was set at a *p* value of less than 0.05.

## 3. Results

**Figure 1:**
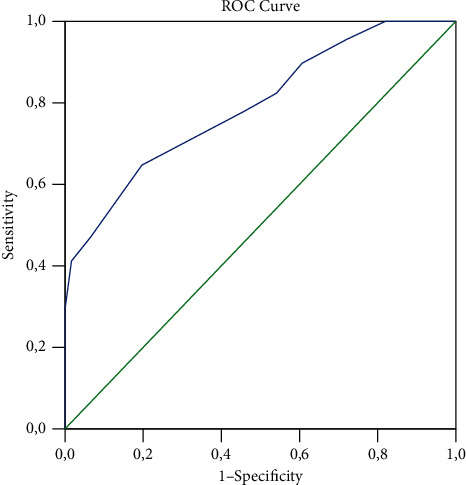
Receiver operating characteristic curve (ROC) analysis representing the curve obtained to differentiate patients with noncomplicated appendicitis from patients with complicated appendicitis (AUC = 0.793, 95% CI: 0.719–0.868, *p* < 0.001).

Among the patients that underwent appendectomy during the study period, a total of 129 patients were identified and included in this study. Recorded data showed that 68 (52.7%) patients were found to have CA and the remaining 61 (47.3%) patients had NCA. The majority of female patients were found with CA (40, 65.6%) rather than NCA (21, 34.4%). On the contrary, more men presented with NCA (40, 58.8%), data suggesting that significantly more female patients presented with CA (*p*=0.006). The mean age of the patients in the CA group was significantly higher than that of the NCA group (*p*=0.017), demonstrating that more severe appendicitis tends to occur in older patients. The most common preoperative presentations of patients in both groups are given in Table 1 as well as major postoperative complications, namely, superficial surgical site infections, deep surgical site infections-intraabdominal abscess, and systemic complications (pulmonary and urinary infections). Apart from the preoperative finding of abdominal guarding that was found more frequently in patients of the CA group (*p* < 0.001), no significant differences were found between the two groups in both presentation forms and postoperative complications. Although the majority of the patients of both groups had normal preoperative sodium levels, hyponatremia was found significantly more frequently in patients with CA than in patients with NCA (41.2% vs. 1.6% respectively, *p* < 0.001) (Table 1). In 38 out of 68 (55.9%) patients with CA, no evidence of appendiceal perforation was found, and in 30 patients (44.1%), perforation was documented. The ROC curve of serum sodium levels upon admission identifying patients with CA showed an AUC of 0.793 (95% CI: 0.718–0.868) (Figure 1). The cutoff value of ≤135 mEq/L, which in our study was used to distinguish patients with hyponatremia, showed sensitivity 41.4%, specificity 98.3%, positive predictive value 96.6%, and negative predictive value 60%. Comparison of the perforation–no perforation subgroups of patients with CA showed that no significant difference (*p*=0.376) of the preoperative sodium levels was found between the two groups. When all patients included in the study were classified into the perforation–no perforation subgroups, comparison revealed that significantly more patients with perforation (36.7%) were found to have low preoperative sodium levels than patients without perforation (19.2%) (*p*=0.047).

## 4. Discussion

The present study demonstrated that preoperative hyponatremia was found more frequently in patients that proved to have CA than in those with NCA and also more frequently in patients with documented perforation than without perforation. Since CA is usually associated with unfavorable outcome, identification of preoperative hyponatremia, a low-cost, routine laboratory examination, could indicate increased possibility of appendiceal gangrene and/or perforation. This finding could potentially affect the course of patient management, leading to earlier operative intervention and abandonment of observational or nonoperative strategy.

Hyponatremia has been associated with poor prognosis and increased length of hospital stay in a variety of clinical situations including community-acquired pneumonia and spontaneous bacterial peritonitis in liver cirrhosis [16–18]. In the surgical setting, it has been demonstrated that preoperative hyponatremia is associated with increased perioperative morbidity and mortality [13]. Hyponatremia at admission was found helpful in both establishing the diagnosis of necrotizing soft-tissue infection as well as in predicting mortality in those patients [19, 20]. Among various admission variables, hyponatremia was identified as a potential predictor of gangrenous cholecystitis and bowel ischemia and/or perforation in small bowel obstruction [21, 22].

There is an increasing body of evidence suggesting that hyponatremia is associated with visceral wall ischemia and/or perforation. According to Swart et al. [15], the clinical association between a severe inflammatory stimulus and hyponatremia can be explained by the so-called “immuno-neuroendocrine interface,” in which interleukin-6 (IL-6) plays an important role. During inflammation, several proinflammatory cytokines, including IL-6, are secreted initiating the acute phase response [23]. Circulating IL-6 is either transported or simply diffuse across the blood-brain barrier activating the subfornical organ and organum vasculosum of the lamina terminalis [24]. This activation eventually leads to increased vasopressin secretion by neurons of supraoptic and paraventricular nuclei and thirst. Subsequently, the combination of antidiuresis caused by cytokine-mediated nonosmotic vasopressin secretion with increased water intake results in hyponatremia [15].

Previous efforts to evaluate a possible relation between hyponatremia and the severity of appendicitis have been reported in both adult and pediatric populations. In a retrospective study involving 1550 adults with acute appendicitis, Kim et al. [25] investigated the association between various clinical and laboratory parameters with intraoperatively identified perforated or gangrenous appendicitis and found that hyponatremia could be suggestive of complicated appendicitis. Käser et al. [11] evaluated hyponatremia as a marker of colon perforation in sigmoid diverticulitis or appendicitis in patients older than 50 years and concluded that hyponatremia can be considered as such a marker, but since it has low sensitivity, the absence of hyponatremia cannot predict the absence of colon perforation. However, the authors do not clearly state whether the perforated appendicitis subgroup showed the same association with hyponatremia as the total of patients with bowel perforation [11]. In an effort to assess the clinical characteristics, risk factors, and prognosis of acute appendicitis in adults undergoing hemodialysis, Wu et al. [26] found that appendiceal perforation was present in 66% of the patients with preoperative hyponatremia but without significant difference with the nonhyponatremia group.

The association between hyponatremia and the severity of appendicitis was also investigated in pediatric populations. In a prospective diagnostic accuracy study including 80 children with acute appendicitis, Lindestam et al. [27] confirmed that there is a strong association between plasma sodium concentration ≤136 mmol/L and appendiceal perforation. Pogorelic et al. [28], in a study including 184 pediatric patients, reported that the sodium concentration cutoff value of ≤135 mmol/L was shown to give the best possible sensitivity (94.7%) and specificity (88.5%), also confirming hyponatremia as a promising new biochemical marker indicating complicated appendicitis. Besli et al. [28] in a retrospective case control study found no difference in sodium levels between children with and without complicated appendicitis. This study also reported that patients with CA had lower baseline sodium levels, with the cutoff level for basal Na ≤138 mEq/L providing a sensitivity of 82.5% and specificity of 31.1% [29]. Similar retrospective studies by Pham et al. [12] and Serradilla et al. [30] both concluded that hyponatremia was significantly associated with CA. In a systematic review including 7 studies from both pediatric and adult populations, Giannis et al. [31] concluded that serum sodium level measurement should be taken into consideration in patients suspected of having complicated acute appendicitis.

Limitations of the present study include its retrospective design and the relatively small sample size, mainly due its single-institution design as well as to a small number of missing data, affecting the power of the study. Another limitation arises from the discrepancies between various studies concerning the cutoff value used for the serum sodium levels. In our study, hyponatremia was defined as a serum sodium level of ≤135 mEq/L, while other studies arbitrarily used <135 mEq/L, ≤136 mEq/L, or <136 mEq/L, making comparison of the results less reliable.

## 5. Conclusions

In conclusion, our study demonstrated that preoperative hyponatremia is associated with CA. Serum sodium levels, a routine, low-cost laboratory test, could act as an accessory marker aiding surgeons in earlier identification of gangrenous or perforated acute appendicitis. Future prospective studies will further clarify the relation between the severity of appendicitis and hyponatremia leading to optimal clinical management.

## Figures and Tables

**Table 1:** Patient classification in noncomplicated appendicitis and complicated appendicitis according to sex (Pearson's *x*^2^ test), age (Student's *t*-test), preoperative presentation (abdominal guarding, nausea/vomiting, and fever—Pearson's *x*^2^ test and peritonitis—Fisher's exact test), postoperative complications (superficial SSI, deep SSI, and systematic—Fisher's exact test), and preoperative sodium levels (Fischer's exact test).

Patient demographics		NCA, *n* = 61	CA, *n* = 68	*P*
Sex	Male, *n* (%)	40 (58.8)	28 (41.2)	0.006
	Female, *n* (%)	21 (34.4)	40 (65.6)	
Age	Mean (SD)	33.8 (14.7)	45.8 (21.5)	0.017
Preoperative presentation	Abdominal guarding	20 (32.8)	48 (70.6)	<0.001
	Peritonitis	0 (0%)	4 (5.8%)	NS
	Nausea/vomiting	28 (45.9%)	32 (47.1%)	NS
	Fever	7 (11.5%)	10 (14.7%)	NS
Preoperative sodium levels	Normonatremia	60 (98.4%)	40 (58.8%)	0.001
	Hyponatremia	1 (1.6%)	28 (41.2%)	
Postoperative complications	Superficial SSI	3 (4.9%)	7 (10.3%)	NS
	Deep SSI abscess	1 (1.6%)	4 (5.8%)	NS
	Systematic	1 (1.6%)	5 (7.3%)	NS

NCA, noncomplicated appendicitis; CA, complicated appendicitis; SD, standard deviation; SSI, surgical site infection; NS, nonsignificant.

## Data Availability

The data (nominal and ordinal) used to support the findings of this study are available from the corresponding author upon request.

## References

[1] Debnath J., Kumar R., Mathur A. (2015). On the role of ultrasonography and CT scan in the diagnosis of acute appendicitis. *Indian Journal of Surgery*.

[2] Nshuti R., Kruger D., Luvhengo T. E. (2014). Clinical presentation of acute appendicitis in adults at the chris hani baragwanath academic hospital. *International Journal of Emergency Medicine*.

[3] Prystowsky J. B., Pugh C. M., Nagle A. P. (2005). Current problems in surgery. appendicitis. *Current Problems in Surgery*.

[4] Lee J. F. Y., Leow C. K., Lau W. Y. (2000). Appendicitis in the elderly. *Australian and New Zealand Journal of Surgery*.

[5] Romano A., Parikh P., Byers P., Namias N. (2014). Simple acute appendicitis versus non-perforated gangrenous appendicitis: is there a difference in the rate of post-operative infectious complications?. *Surgical Infections*.

[6] Ponsky T. A., Huang Z. J., Kittle K. (2004). Hospital- and patient-level characteristics and the risk of appendiceal rupture and negative appendectomy in children. *JAMA*.

[7] Franz M. G., Norman J., Fabri P. J. (1995). Increased morbidity of appendicitis with advancing age. *The American Surgeon*.

[8] Pogorelic Z., Domjanovic J., Jukic M., Poklepovic-Pericic T. (2020). Acute appendicitis in children younger than five years of age: diagnostic challenge for pediatric surgeons. *Surgical Infections*.

[9] Symer M. M., Abelson J. S., Sedrakyan A., Yeo H. L. (2018). Early operative management of complicated appendicitis is associated with improved surgical outcomes in adults. *The American Journal of Surgery*.

[10] Pogorelic Z., Mihanovic J., Nincevic S., Luksic B., Baloevic S. E., Polasek O. (2021). Validity of appendicitis inflammatory response score in distinguishing perforated from non-perforated appendicitis in children. *Children*.

[11] Käser S. A., Furler R., Evequoz D. C., Maurer C. A. (2013). Hyponatremia is a specific marker of perforation in sigmoid diverticulitis or appendicitis in patients older than 50 years. *Gastroenterology Research and Practice*.

[12] Pham X.-B. D., Sullins V. F., Kim D. Y. (2016). Factors predictive of complicated appendicitis in children. *Journal of Surgical Research*.

[13] Leung A. A., McAlister F. A., Rogers S. O., Pazo V., Wright A., Bates D. W. (2012). Preoperative hyponatremia and perioperative complications. *Archives of Internal Medicine*.

[14] Park S. J., Shin J. I. (2013). Inflammation and hyponatremia: an underrecognized condition?. *Korean Journal of Pediatrics*.

[15] Swart R. M., Hoorn E. J., Betjes M. G., Zietse R. (2011). Hyponatremia and inflammation: the emerging role of interleukin-6 in osmoregulation. *Nephron Physiology*.

[16] Thompson C., Hoorn E. J. (2012). 1 Hyponatraemia: an overview of frequency, clinical presentation and complications. *Best Practice & Research Clinical Endocrinology & Metabolism*.

[17] Nair V., Niederman M. S., Masani N., Fishbane S. (2007). Hyponatremia in community-acquired pneumonia. *American Journal of Nephrology*.

[18] Sigal S. H. (2012). Hyponatremia in cirrhosis. *Journal of Hospital Medicine*.

[19] Wong C.-H., Khin L.-W., Heng K.-S., Tan K.-C., Low C.-O. (2004). The LRINEC (laboratory risk indicator for necrotizing fasciitis) score: a tool for distinguishing necrotizing fasciitis from other soft tissue infections^∗^. *Critical Care Medicine*.

[20] Yaghoubian A., de Virgilio C., Dauphine C., Lewis R. J., Lin M. (2007). Use of admission serum lactate and sodium levels to predict mortality in necrotizing soft-tissue infections. *Archives of Surgery*.

[21] Falor A. E., Zobel M., Kaji A., Neville A., de Virgilio C. (2012). Admission variables predictive of gangrenous cholecystitis. *The American Surgeon*.

[22] O’Leary M. P., Neville A. L., Keeley J. A., Kim D. Y., de Virgilio C., Plurad D. S. (2016). Predictors of ischemic bowel in patients with small bowel obstruction. *The American Surgeon*.

[23] Gabay C., Kushner I. (1999). Acute-phase proteins and other systemic responses to inflammation. *New England Journal of Medicine*.

[24] Banks W. A., Kastin A. J., Gutierrez E. G. (1994). Penetration of interleukin-6 across the murine blood-brain barrier. *Neuroscience Letters*.

[25] Kim D. Y., Nassiri N., de Virgilio C. (2015). Association between hyponatremia and complicated appendicitis. *JAMA Surgery*.

[26] Wu H.-C., Yan M.-T., Lu K.-C. (2013). Clinical manifestations of acute appendicitis in hemodialysis patients. *Surgery Today*.

[27] Lindestam U., Almström M., Jacks J. (2020). Low plasma sodium concentration predicts perforated acute appendicitis in children: a prospective diagnostic accuracy study. *European Journal of Pediatric Surgery*.

[28] Pogorelic Z., Luksic B., Nincevic S., Luksic B., Polasek O. (2021). Hyponatremia as a predictor of perforated acute appendicitis in pediatric population: a prospective study. *Journal of Pediatric Surgery*.

[29] Besli G. E., Cetin M., Ulukaya Durakbasa C., Ozkanli S. (2019). Predictive value of serum sodium level in determining complicated appendicitis risk in children. *Haydarpasa Numune Training Research Hospital Medical Journal*.

[30] Serradilla J., Bueno A., De la Torre C. (2018). Predictive factors of gangrenous post-appendectomy intra-abdominal abscess. A case-control study. *Cirugía Pediátrica*.

[31] Giannis D., Matenoglou E., Moris D. (2020). Hyponatremia as a marker of complicated appendicitis: a systematic review. *The Surgeon*.

